# Regulation of somatostatin receptor 2 in the context of antidepressant treatment response in chronic mild stress in rat

**DOI:** 10.1007/s00213-018-4912-x

**Published:** 2018-04-30

**Authors:** Agata Faron-Górecka, Maciej Kuśmider, Joanna Solich, Magdalena Kolasa, Paulina Pabian, Piotr Gruca, Irena Romańska, Dariusz Żurawek, Marta Szlachta, Mariusz Papp, Lucyna Antkiewicz-Michaluk, Marta Dziedzicka-Wasylewska

**Affiliations:** 1 Department of Pharmacology, Laboratory of Biochemical Pharmacology, Institute of Pharmacology, Polish Academy of Sciences, Smętna Street 12, 31-343 Kraków, Poland; 2 Department of Pharmacology, Laboratory of Behavioral Pharmacology, Institute of Pharmacology, Polish Academy of Sciences, Smętna Street 12, 31-343 Kraków, Poland; 3 Department of Neurochemistry, Institute of Pharmacology, Polish Academy of Sciences, Smętna Street 12, 31-343 Kraków, Poland

**Keywords:** Chronic mild stress, sst2R, D2R, Dopamine level, Medial habenula nucleus, Autoradiography

## Abstract

**Rationale:**

The role of somatostatin and its receptors for the stress-related neuropsychiatric disorders has been widely raised. Recently, we have also demonstrated the involvement of somatostatin receptor type 2-sst2R and dopamine receptor type 2-D2R in stress.

**Objective:**

In this context, we decided to find if these receptors are involved in response to antidepressant treatment in animal model of depression—chronic mild stress (CMS).

**Methods:**

Here, we report data obtained following 7-week CMS procedure. The specific binding of [125I]Tyr3-Octreotide to sst2R and [3H]Domperidone to D2R was measured in the rat brain, using autoradiography. Additionally, the level of dopamine and metabolites was measured in the rat brain.

**Results:**

In the final baseline test after 7 weeks of stress, the reduced consumption of sucrose solution was observed (controls vs the stressed animals (6.25 0.16 vs. 10.39 0.41; *p* < 0.05). Imipramine was administered for the next 5 weeks, and it reversed anhedonia in majority of animals (imipramine-reactive); however, in some animals, it did not (imipramine-non-reactive). Two-way repeated measures ANOVA revealed significant effects of stress and treatment and time interaction [*F*(16, 168) = 3.72; *p* < 0.0001], *n* = 10 per groups. We observed decreased binding of [125I]Tyr3-Octreotide in most of rat brain regions in imipramine non-reactive groups of animals. The decrease of D2R after stress in striatum and nucleus accumbens and no effect of imipramine were observed.

In the striatum and prefrontal cortex, the significant role of stress and imipramine in dopamine levels was observed.

**Conclusions:**

The results obtained in binding assays, together with dopamine level, indicate the involvement of sst2R receptors for reaction to antidepressant treatment. Besides, the stress context itself changes the effect of antidepressant drug.

## Introduction

Affective disorders, including depression, are often associated with the dysregulation of neuropeptides in various brain regions. One of these neuropeptides is somatostatin (SST). SST and its receptors (five somatostatin receptor subtypes sst_1_R-sst_5_R) are widely distributed across the central nervous system. SST receptors are G-protein-coupled receptors that are responsible for the inhibition of adenylate cyclase, activation of potassium channels, and stimulation of tyrosine kinase (Hoyer et al. 1995). Pathophysiology in the action of SST along with other neuromodulating systems has been implicated in depression (Pallis et al. 2001; Faron-Górecka et al. 2013). The involvement of SST dysregulation in affective disorders was suggested due to the low concentration of SST recorded in the cerebrospinal fluid of patients with depression (Molchan et al. 1991; Frye et al. 2003). Recently, it has been demonstrated that SST-positive GABAergic interneurons are involved in the pathology of major depression, with a reduced expression of SST observed in the post mortem brains of patients (Guilloux et al. 2012; Sibille et al. 2011). It has been demonstrated that SST is downregulated at the mRNA level and at the precursor protein level in the anterior cingulate cortex and dorsolateral prefrontal cortex of depression patients (Tripp et al. 2011; Sibille et al. 2011). Disinhibition of somatostatin-positive GABAergic interneurons through the use of SSTCre mice results in an anxiolytic and antidepressant-like brain state (Fuchs et al. 2017). Because the striatum and nucleus acumbens (NAcc) are reported to contain both SST and its receptors, it is also possible that SST regulates dopaminergic function in these brain areas (Ikeda et al. 2012). Chronic antidepressant treatment influences the effects of SST on dopamine function selectively in the NAcc (Pallis et al. 2001). SST mediates various physiological and behavioural actions by interacting with multiple somatostatin receptor subtypes (Hoyer et al. 1995). In the NAcc, the sst1 receptor (sst_1_R) has been reported to be an autoreceptor for SST (Vasilaki et al. 2004; Thermos et al. 2006), whereas the sst2 receptor (sst_2_R) appears to be responsible for the actions of SST on dopamine release and dopamine-mediated behaviours (Thermos et al. 1996; Hathway et al. 1999). The role of sst_2_R in emotional processes, such as anxiety or depression, is well recognised (Engin and Treit 2009). It has been demonstrated that sst_2_-knock out (KO) mice have high corticosterone levels and display anxiety-like behaviours, while both sst_2_KO and sst_4_KO mice exhibit an antidepressant-like effect (Prévôt et al. 2017). Additionally, the increased expression of the mRNA-encoding sst_2_R within the amygdala and anterior cingulate cortex in the predator stress model has been observed (Nanda et al. 2008). In our previously published work, we demonstrated the involvement of sst_2_R in response to 2 weeks of chronic unpredictable stress (CMS) in rats (Faron-Górecka et al. 2016).

The full procedure of CMS allows for the identification of a specific group of animals that do not respond behaviourally to imipramine (IMI) treatment. Because our previous studies indicated a role of sst_2_R in the stress response, we decided to examine the role of sst_2_R in the context of the antidepressant treatment response. Additionally, because an interaction between the somatostatin and dopamine systems has been postulated (Pallis et al. 2001), we decided to examine how CMS affects the binding of dopamine D_2_ receptors and the DA level.

## Materials and methods

### Animals

Male Wistar Han rats were purchased from Charles River, Germany. The weight of the animals was nearly 300 g when the adaptation of sucrose consumption was initiated and approximately 350 g at the start of stress procedure. Rats were brought into the laboratory 1 month prior to the start of the behavioural and biochemical experiments. Except when grouping was applied as a stress parameter, they were singly housed in plastic cages (40 × 25 × 15 cm). Food and water were provided ad libitum, except when food or/and water deprivation was applied as a stress parameter. The standard 12-h light/dark cycle was maintained, except during the course of the stress regime. This study was approved by the Bioethical Committee at the Institute of Pharmacology at the Polish Academy of Sciences, Krakow, Poland.

### Sucrose consumption test

Prior to the stress experiments, the animals were trained to consume a sucrose solution (1%). The training procedure lasted for 6 weeks and consisted of 1-h testing sessions every week, in which the sucrose solution was presented to the rats in their home cages after 14 h of food and water deprivation. Sucrose intake was measured after each drinking test as the difference in the weight of the bottle. During the 7 weeks of stress protocol, the sucrose consumption test was performed once a week. The operational cut-off point between the control and stress-reactive group was based on an arbitrary retrospective observation that was set to be a sucrose consumption of 7.5 g.

Anhedonic and IMI non-reactive (IMI-NR) animals displayed sucrose consumption that was lower (below 7.5 g) than the final baseline test. Animals reactive to IMI (IMI-R) administration demonstrated an increase in sucrose intake to above 7.5 g.

### Chronic mild stress protocol

CMS experiments were performed according to the method that has been described previously (Żurawek et al. 2015; Faron-Górecka et al. 2014). Each week of the stress regime consisted of two periods of food or water deprivation; two periods of 450 cage tilt; two periods of intermittent illumination (lights on and off every 2 h); two periods of soiled cage (250 ml water in sawdust bedding); two periods of paired housing; two periods of low-intensity stroboscopic illumination (150 flashes/min); and two periods of no stress stimuli. All stressors were presented for 10–14 h and were applied individually and continuously, day and night. Animals were deprived of food and water for 14 h preceding each sucrose test, but otherwise food and water were freely available in the home cage. Control animals remained undisturbed in a separate room with free access to food and water, except for a period of overnight deprivation prior to the sucrose consumption test once a week. On the basis of their sucrose intake in the final baseline test, animals were subjected to the CMS procedure for 7 weeks. After the second week of the stress procedure, the groups of animals started to receive IMI administration for the next 5 weeks (10 mg/kg b.w.). Control animals received daily injections of a vehicle (sterile saline, 1 ml/kg b.w.). The weekly sucrose tests were performed 24 h following the final injection of drug or vehicle. Stress conditions were continued throughout the entire period of treatment.

### Drug administration

Drug and vehicle administration was performed daily in the morning at approximately 10.00 a.m. IMI (Sigma Aldrich, Germany) was dissolved in physiological saline and was administered at a dose of 10 mg/kg, i.p.

### Tissue preparation

The rats were sacrificed by decapitation 24 h after the final sucrose test. The brains were rapidly removed and frozen using a heptane-dry ice mixture. Coronal brain sections (12 μm) were cut using a Jung CM 3000 cryostat microtome (Leica, Germany). The slices were thaw mounted on gelatine-covered microscope slides, air dried, and stored at − 20 °C until use. For measurements of the level of dopamine and its metabolites, the appropriate brain regions were dissected out of the removed rat brain and immediately frozen on dry ice.

### Somatostatin receptor autoradiography: binding of Tyr^25^[^125^I]-Leu^8^, D-Trp^22^

Receptor autoradiography was performed as described by Ferone et al. 1999. Briefly, slide sections were preincubated for 10 min in 170 mM Tris-HCl buffer, pH 7.4, and then incubated for 60 min at room temperature (RT) in 170 mM Tris-HCl buffer containing 5 mM MgCl_2_ and 1% bovine serum albumin with 0.1 nM Tyr^25^, [^125^I]-Leu^8^, and D-Trp^22^ (Perkin Elmer, Germany). Non-specific binding was determined using 1 μM SST14 non-labelled rat somatostatin-14 (Prospec, Israel). After incubation, slides were dipped twice in ice-cold Tris-HCl buffer and in ice-cold deionised H_2_O. Finally, the sections were dried under a stream of cold air. Radiolabelled sections were exposed to Kodak Biomax XAR film (Sigma-Aldrich) for 7 days. Autoradiography images were digitised and quantified using the MCID System. Brain regions were identified according to the rat brain atlas of Paxinos and Watson (1986).

### Dopamine D_2_ receptor autoradiography

Dopamine D2 receptor autoradiography was performed according to our previous publication (Żurawek et al. 2013). The rat brain sections were pre-incubated in 50 mM Tris-HCl buffer (pH 7.4) at RT for 15 min to remove endogenous dopamine. The brain slices were then incubated for 2 h at RT in 50 mM Tris-HCl (pH 7.4) containing 120 mM NaCl, 1 mM EDTA, 1.5 mM CaCl_2_, 4 mM MgCl_2_, and 5 mM KCl with 0.4 nM [^3^H]domperidone. To determine non-specific binding, slices were treated with 10 μM (+)butaclamol and incubated in the same binding buffer that was described previously. After incubation, slides were dipped twice in ice-cold Tris-HCl buffer and in ice-cold deionised H_2_O. The sections were dried overnight under a stream of air. The labelled brain slices were exposed to an imaging plate (Fujifilm, Japan) with autoradiography microscales (GE Healthcare) for 7 days. The resulting autoradiograms were analysed and quantified using ImageGauge software (Fujifilm, Japan).

### Levels of DA and its metabolites: high-performance liquid chromatography

Dopamine (DA) and its metabolites, 3,4-dihydroxyphenylacetic acid (DOPAC) and 3-methoxytyramine (3-MT), and the final metabolite, homovanillic acid (HVA), were assayed using high-performance liquid chromatography with electrochemical detection, under the conditions described by Wasik et al. (2007). The tissue samples were weighed and homogenised in ice-cold 0.1 M perchloroacetic acid containing 0.05 mM ascorbic acid. After centrifugation (10,000×*g*, 5 min), the supernatants were filtered through RC 58 0.2-im cellulose membranes (Bioanalytical Systems, West Lafayette, IN, USA). The chromatograph HP1050 (Hewlett-Packard, Golden, CO, USA) was equipped with C18 columns. The mobile phase consisted of 0.05 M citrate-phosphate buffer, pH 3.5, 0.1 mM EDTA, 1 mM sodium octyl sulfonate, and 3.5% methanol. The flow rate was maintained at 1 ml/min. The chromatographic data were processed using the ChemStation computer program (Hewlett Packard USA) and dopamine and its metabolites were quantified through peak height comparisons with standards run on the day of analysis.

### Data quantification and statistical analysis

Behavioural data after 7 weeks of stress protocol and 5 weeks of IMI treatment were analysed using two-way ANOVA repeated measures with stress protocol and IMI treatment as the between subject factors and time as the within subject factor using the Bonferroni multiple comparison post hoc test. All groups consisted of 10 animals. Biochemical data were analysed using two-way ANOVA with Bonferroni post hoc test to compare all groups of experiments after 7 weeks of stress protocol and 5 weeks of IMI treatment.

## Results

### Effects of chronic mild stress and imipramine treatment on sucrose consumption

**Fig. 1:**
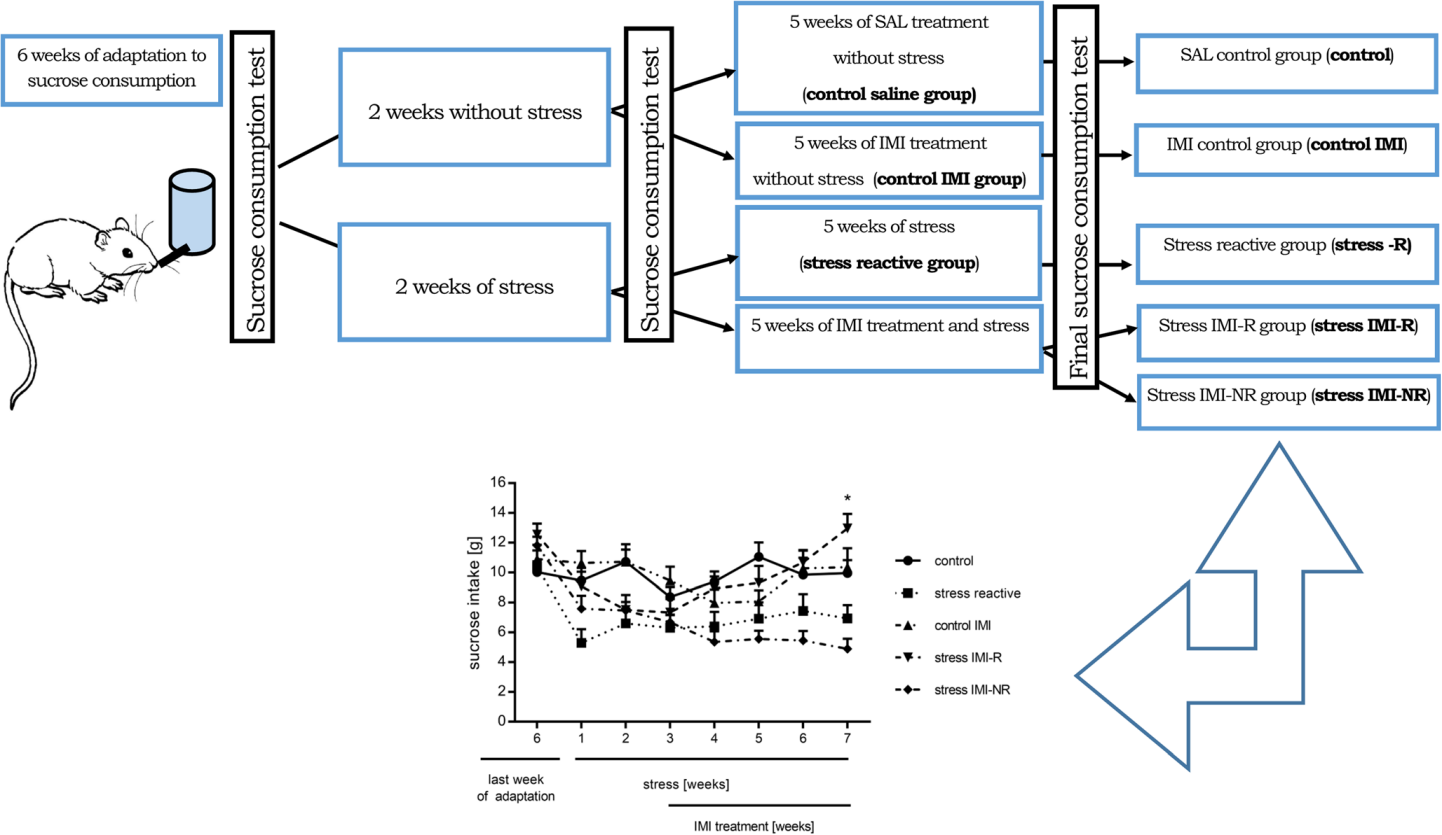
The scheme of full CMS procedure. The graph shows the differences in sucrose intake after full procedure of CMS. Data represents mean ± S.E.M., *n* = 10 animals per group

Independent analyses using repeated measures ANOVA test did not show any significant differences in sucrose consumption among animals during the training procedure in the chronic mild stress experiment [*F*(2, 29) = 3.485; *p* = 0.052]. In the final baseline test after 7 weeks of stress protocol, sucrose intake was significantly different between the control animals and the animals that had been subjected to the stress protocol (6.25 ± 0.16 vs. 10.39 ± 0.41; *p* < 0.05). Administration of IMI for 5 weeks in the group of animals that was still being subjected to the CMS protocol resulted in a significant reduction in anhedonia, as measured by sucrose intake. However, some animals did not respond to IMI treatment in this model of depression (about 20%). Two-way repeated measures ANOVA revealed significant interactions between the stress protocol and IMI treatment with time [*F*(16, 168) = 3.72; *p* < 0.0001]. A significant effect of the stress protocol and IMI treatment [*F*(4, 168) = 9.27; *p* < 0.0001] and time [*F*(4, 168) = 5.25; *p* < 0.01] was observed (Fig. 1). As a result of the data obtained from these experiments, the following groups of animals were selected: control; stressed; control IMI; stress and IMI reactive (IMI-R); stress reactive and IMI non-reactive (IMI-NR; Fig. 1).

### Influence of chronic mild stress and imipramine on the [^125^I]Tyr3-octreotide level in rat brain

**Fig. 2:**
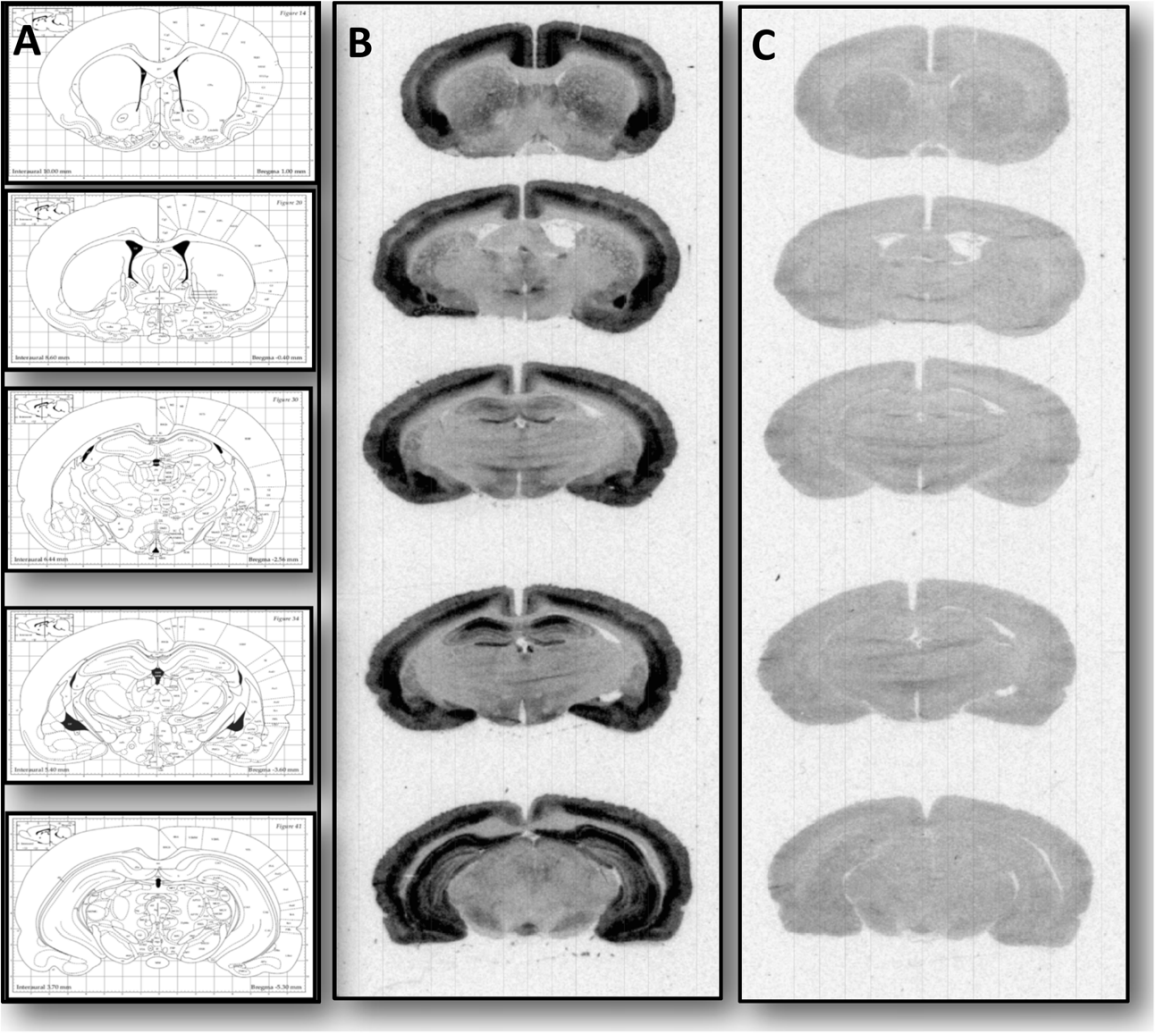
**a** Examined rat brain section based on the rat brain atlas Paxinos and Watson. Representative autoradiograms (**b**) total (**c**) non-specific [^125^I] Tyr^25^,[^125^I]-Leu^8^, D-Trp^22^ ([^125^I]Tyr3-Octreotide) binding

In our studies, we used the somatostatin-28, Tyr25, [^125^I]-Leu8, and D-Trp22 ([^125^I]Tyr3-Octreotide) as a radioligand. This compound is a SST analogue that serves as pharmaceutical octreotide acetate, which has a high affinity to sst_2_R and sst_5_R receptors (Patel 1999). Because sst_5_R mRNA is present at low levels in the adult rodents brain (Hannon et al. 2002; Feuerbach et al. 2000) and sst_5_R is mainly expressed in the rat pituitary (Shimon 2003), we can indirectly say that brain receptor autoradiography using [^125^I]Tyr^3^-Octreotide allowed us to observe mainly sst_2_R binding. Such an assumption is also supported by studies, in which the binding of this radioligand was not detected in SST_2_R knockout mice (Hannon et al. 2002). In agreement with our previous studies (Faron-Górecka et al. 2016), a high density of SST receptors was observed in the control and stressed or/and imipramine treatment groups using [^125^I]Tyr3-Octreotide binding in different brain areas. Representative autoradiograms are presented in Fig. 2. Data obtained in specific brain regions are presented in Table 1. In the majority of brain regions studied, the CMS procedure increased the specific binding of [^125^I]Tyr3-Octreotide. The effect of IMI administration to control, non-stressed animals was similar in the cingulate and primary cortex, as well as in the striatum and NAcc but not in the medial habenular nucleus (MHb) nor in the hippocampus and substantia nigra (SN). Interestingly, a subset of the group of animals subjected to the stress protocol and IMI treatment did not respond to the drug, as measured as a change in sucrose consumption in most brain regions studied (except for hippocampus and hypothalamus); these animals showed significantly lower specific binding of [^125^I]Tyr3-Octreotide than did the stressed group.

**Table 1:** Specific binding of [^125^I] Tyr^25^,[^125^I]-Leu^8^, and D-Trp^22^ in rat brain after CMS procedure. Data represent as optical density (O.D.) ± S.E.M.

Structures	[^125^I] Tyr^25^,[^125^I]-Leu^8^, D-Trp^22^ binding [O.D. ± S.E.M.]				
	Control	Stress	Control IMI	IMI R	IMI NR
Primary cortex	47,206 ± 552	49,943 ± 237^a^	49,603 ± 463	46,407 ± 881	38,349 ± 1917^d,e^
Cingulate cortex	47,277 ± 632	50,194 ± 20 ^a^	47,617 ± 477	47,621 ± 472	40,712 ± 2136^d,e,f^
Dorsal endopiriform nucleus	47,484 ± 538	47,752 ± 582	47,686 ± 592	47,821 ± 536	46,514 ± 633
Striatum, medial part	22,784 ± 1167	29,902 ± 2216 ^a^	28,396 ± 1610	23,815 ± 928	19,078 ± 928
Striatum, lateral part	10,235 ± 839	130,402 ± 1205	13,500 ± 916	11,982 ± 519	7002 ± 896
Accumbens nucleus, core	18,742 ± 1467	25,061 ± 1177 ^a^	24,765 ± 2357	20,151 ± 1295	16,783 ± 1132^d,e^
field Ca1 of hippocampus	47,261 ± 689	50,222 ± 14	45,930 ± 208^b^	47,142 ± 514	43,956 ± 1283^d,f^
dentate gyrus	37,103 ± 2111	40,813 ± 1263	33,590 ± 2079^b^	30,780 ± 1314^c^	32,322 ± 1496^d^
Anterior hypothalamic area, central part	23,190 ± 2904	26,375 ± 5126	21,713 ± 1026	18,817 ± 1594	14,714 ± 1855
Medial habenular nucleus	44,019 ± 605	49,628 ± 426 ^a^	45,353 ± 849	43,143 ± 2275^c^	44,595 ± 644^d^
Molecular layer of the dentate gyrus	47,381 ± 253	50,164 ± 53^a^	45,212 ± 1383^b^	46,766 ± 461^c^	44,703 ± 888^d^
Paraventricular hypothalamic nucleus	25,730 ± 2191	28,394 ± 1401	25,407 ± 1035	25,527 ± 2116	22,365 ± 1332
Paraventricular thalamic nucleus, posterior part	18,921 ± 3224	27,268 ± 2066	26,923 ± 1728	19,544 ± 1687	14,258 ± 2747^d, e^
Basolateral amygdaloid nucleus, anterior part	47,627 ± 496	49,482 ± 620	46,888 ± 624^b^	47,642 ± 415	47,018 ± 645
Medial amygdaloid nucleus, anterodorsal part	46,939 ± 505	49,135 ± 501	40,551 ± 2319^b^	46,082 ± 776	41,482 ± 2275^d^
Interfascicular nucleus	28,075 ± 4990	38,640 ± 3937	31,228 ± 3904	29,517 ± 2862	30,412 ± 1896
Substantia nigra	16,754 ± 954	19,430 ± 907	15,749 ± 837	15,804 ± 984	13,913 ± 706^d^

^a^*p* < 0.05 vs control group; ^b^*p* < 0.05 vs stress group; ^c^*p* < 0.05 vs control IMI group; ^c,d^*p* < 0.05 vs stress group; ^e^*p* < 0.05 vs IMI-R group

An interaction between the stress protocol and IMI treatment was observed in the basal ganglia, primary cortex, MHb, paraventricular hypothalamic nucleus (PVP), and the CA1 of the hippocampus. In the medial striatum, the interaction between the stress protocol and IMI treatment was considered significant [*F*(2, 36) = 12.42; *p* < 0.0001]. Additionally, a statistically significant impact of IMI treatment was observed [*F*(1, 36) = 7.38; *p* < 0.05]. Although, a lack of a statistical significant effect of the stress protocol was observed [*F*(2, 36) = 0.97; *p* > 0.05 (ns)], post hoc analysis revealed a statistically significant difference between the control group (not exposed to the stress protocol) and the stressed group [*p* < 0.05]. In the lateral striatum, the interaction between the stress protocol and IMI treatment was statistically significant [*F*(2, 36) = 10.81; *p* < 0.001], and the effects of individual factors were different to those observed in the medial striatum. A statistically significant impact of stress was observed [*F*(2.36) = 3.334; p < 0.05], while the treatment factor was found to be non-significant [*F*(1.36) = 0.13, ns]. In addition, post hoc statistical analysis showed a significant difference between the IMI-R and IMI-NR group, [*p* < 0.001]. A statistically significant interaction between the stress protocol and IMI treatment was observed in the NAcc [*F*(2, 54) = 13.09; *p* < 0.0001]. The effect of the stress protocol and IMI treatment factors in the NAcc was insignificant [(*F*(2, 54) = 0.66, *F*(1, 54) = 4.00, respectively; ns], although the post hoc statistical analysis showed significant differences between the control and stressed groups [*p* < 0.05]. In the PVP, a significant interaction between the stress protocol and IMI treatment was observed [*F*(2, 24) = 12.73; *p* < 0.0001]; the treatment factor was also statistically significant [*F*(1, 24) = 5.76; *p* < 0.05], while the impact of the stress factor was insignificant [*F*(2, 24) = 0.84; ns]. Moreover, a significant interaction between the stress protocol and IMI treatment was observed in the primary somatosensory cortex [*F*(2, 36) = 27.79; *p* < 0.0001] and a significant impact of stress [*F*(2, 36) = 12.93; *p* < 0.0001] and treatment factors [*F*(1, 36) = 30.46; *p* < 0.0001] was observed. The post hoc analysis revealed a statistically significant difference between the IMI-R and IMI-NR groups [*p* < 0.0001]. Similar changes were revealed in the cingulate cortex (Cg) [interaction, *F*(2, 54) = 14.27; *p* < 0.0001; stress factor *F*(2, 54) = 6.74; *p* < 0.01; treatment factor *F*(1, 54) = 25.65; *p* < 0.0001]. The post hoc analysis showed significant changes between the IMI-R and stress IMI-NR groups [*p* < 0.0001]. In the CA1 of the hippocampus, the interaction between the stress and IMI treatment was statistically significant [*F*(2, 36) = 6.81; *p* < 0.01] and the stress and treatment factors also revealed statistical differences [stress factor, *F*(2, 36) = 5.17; *p* < 0.05; treatment factor, *F*(1, 36) = 41.34; *p* < 0.0001]. The post hoc analysis showed a significant difference between the IMI-R and IMI-NR groups [*p* < 0.05]. In another part of hippocampus, the dentate gyrus (DG), a significant impact of IMI treatment was observed, while the other parameters were not significant [interaction stress × treatment *F*(2, 51) = 2.26; ns; stress factor *F*(2, 51) = 0.29; ns; treatment factor *F*(1, 51) = 31.57; *p* < 0.0001]. The post hoc analysis showed significant differences between the stressed animals (stress group) and the IMI-R [*p* < 0.001] as well as between the stressed group and the IMI-NR group [*p* < 0.01]. A significant effect of the stress protocol [*F*(2, 42) = 5.26; *p* < 0.01] and IMI treatment [*F*(1, 42) = 45.36; *p* < 0.0001] was observed in the molecular layer of the dentate gyrus (Mol), while the interaction of stress and treatment was insignificant [*F*(2, 42) = 3.09; ns]. The post hoc analysis revealed a significance difference between the stressed animals (stress group) and the IMI-R group [*p* < 0.0001]. A significant interaction between the stress and IMI treatment was observed in the MHb [*F*(2, 48) = 7.36; *p* < 0.01]. In this structure, a statistically significant impact of IMI treatment was observed [*F*(1, 48) = 14.72; *p* < 0.001], while the effect of the stress factor was not significant [*F*(2, 48) = 2.64; ns]. However, post hoc analysis revealed a statistically significant difference between the control and stressed groups [*p* < 0.05]. Similar data were obtained for the SN, where the interaction between the stress protocol and IMI treatment was significant [*F*(2, 51) = 3.43; *p* < 0.05], while the effect of the stress factor was not significant [*F*(2, 51) = 1.31; ns] and the effect of IMI treatment was considered to be significant [*F*(1, 51) = 22.94; *p* < 0.0001]. The lack of a significant interaction between the stress and IMI treatment was observed in the anterodorsal part of the medial amygdaloid nucleus (MeAD) [interaction, *F*(2, 36) = 1.40; ns], while the treatment factor was highly significant [treatment factor, *F*(1, 36) = 24.20; *p* < 0.0001] and the impact of stress was also statistically significant [stress factor, *F*(2, 36) = 3.75; *p* < 0.05]. For the anterior part of the basolateral amygdaloid nucleus (BLA), only a significant impact of IMI treatment was observed [*F*(1, 30) = 10.46; *p* < 0.01]. For the other parameters, two-way ANOVA analyses did not show differences of statistical significance [interaction stress × treatment (*F*(2, 30) = 0.94; ns; stress factor *F*(2, 30) = 2.29; ns]. Both for the central part of the anterior hypothalamic area (AHC) and the posterior part of the paraventricular thalamic nucleus (PA), a significant effect of IMI treatment was observed [*F*(1, 18) = 5.80; *p* < 0.05; *F*(1, 36) = 4.64; *p* < 0.05, respectively]. There were no statistically significant effects of the remaining parameters [AHC: interaction *F*(2, 18) = 0.3648; ns; stress factor *F*(2, 18) = 0.21; ns; and PA: interaction *F*(2, 36) = 1.34; ns; stress factor *F*(2, 36) = 0.48; ns]. Data obtained in the dorsal endopiriform nucleus (Den) and the interfascicular nucleus (IF) did not show any statistical differences [Den: interaction *F*(2, 48) = 0.99; ns; stress factor *F*(2, 48) = 0.70; ns; treatment factor *F*(1, 48) = 0.49; ns; IF: interaction *F*(2, 30) = 1.45; ns, stress factor *F*(2, 30) = 0.91; ns, treatment factor *F*(1, 30) = 2, 08; ns].

### Effect of chronic mild stress on dopamine D_2_R expression

**Fig. 3:**
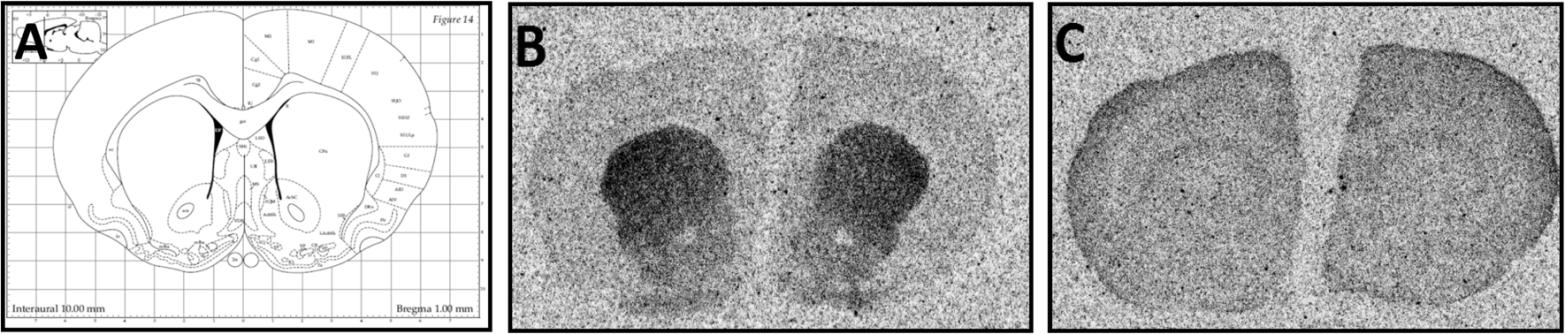
**a** Examined rat brain section based on the rat brain atlas Paxinos and Watson. Representative autoradiograms (**b**) total (**c**) non-specific [^3^H]Domperidone binding

**Fig. 4:**
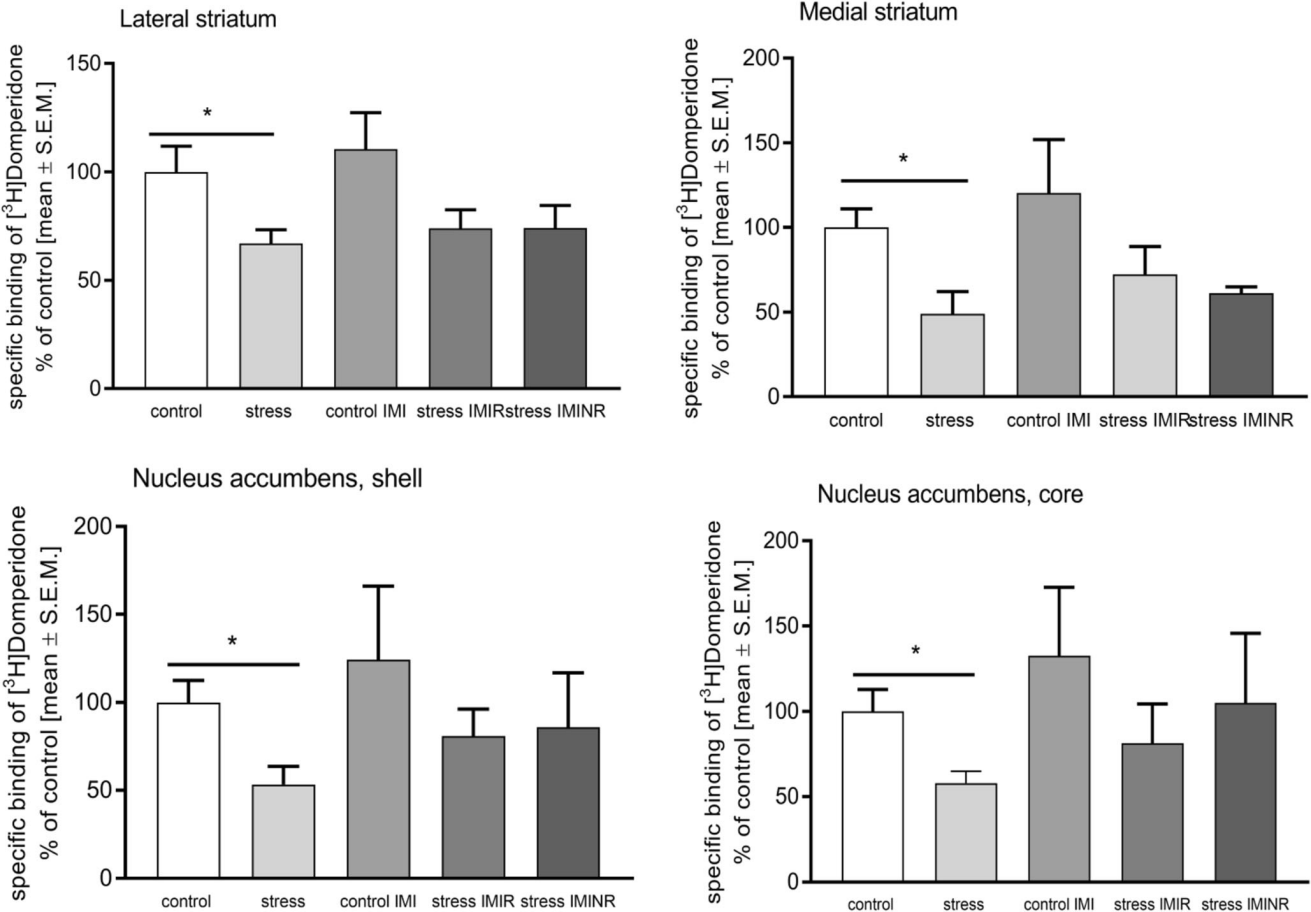
Specific binding of [^3^H]Domperidone. Data was normalised to % of control and represented as mean ± S.E.M.

For the dopamine D_2_ receptor binding, we used [^3^H]Domperidone. The results are presented in Fig. 4. Specific binding was observed in the striatum (lateral and medial parts) and in the NAcc and nucleus accumbens shell (NAcs; Fig. 3). After 7 weeks of stress, a decrease in specific binding in all labelled structures was observed. A statistically significant impact of stress was observed in the lateral part of striatum [*F*(1, 24) = 11.91; *p* < 0.01], while the effect of IMI treatment was not significant [*F*(2, 24) = 0.33; ns]. Similarly, in the medial part of the striatum, a statistically significant effect of the stress was observed [*F*(1, 21) = 8.14; *p* < 0.01]. Post hoc analysis revealed a statistically significant difference between control and stressed groups [*p* < 0.05]. However, the two-way ANOVA did not show any statistically significant changes. In the NAcc and NAcs, the stress protocol induced a significant decrease in the binding of [^3^H]Domperidone [*p* < 0.05] (Fig. 4).

### Effect of CMS on DA and its metabolites in the PFC, striatum, and hypothalamus

Our results indicate a significant role of stress in the levels of DA in the PFc and striatum, with a statistically significant effect of stress on the level of 3-MT in the striatum and an interaction between stress and IMI treatment (Table 2). In the PFc, the impact of stress on the level of DA was considered to be significant [*F*(1, 18) = 20.87; *p* < 0.001], despite the lack of a statistically significant effect of IMI treatment [*F*(2, 18) = 0.86; ns]. Similar changes were observed in the level of DOPAC [impact of stress *F*(1, 18) = 5.71; *p* < 0.05; impact of treatment *F*(2, 18) = 1.23; ns]. As far as the levels of 3-MT and HVA are concerned, a statistically significant impact of IMI treatment was observed [*F*(2, 18) = 6.58; *p* < 0.01 and *F*(2, 18) = 3.60; *p* < 0.05, for 3-MT and HVA respectively]. A statistically significant impact of stress was observed in the DA level in the striatum [*F*(1, 18) = 6.573; *p* < 0.05]. Two-way ANOVA analysis revealed an interaction between stress and IMI treatment [*F*(2, 18) = 5.622; *p* < 0.05] and a statistically significant effect of stress on the level of 3-MT [*F*(1, 18) = 11.79; *p* < 0.01]. For other metabolites (DOPAC and HVA), analysis did not show any significant effects. In the hypothalamus, we did not observe any significant changes in the levels of DA, 3-MT, or HVA. The impact of stress in the hypothalamus produced a significant change in the level of DOPAC only [*F*(1, 18) = 5.84, *p* < 0.05].

**Table 2:** Dopamine and metabolites levels in rat brain after CMS procedure

(a) DA and metabolites levels in PFc					
PFc—7 weeks of stress and 5 weeks of imipramine treatment					
Treatment	*N*	[DA]	[DOPAC]	[3-MT]	[HVA]
Control	4	1450 ± 199	310 ± 35	60 ± 9.1	155 ± 19
Stress reactive	4	1669 ± 130	383 ± 35	65 ± 9.3	190 ± 35
Control IMI	4	1269 ± 137	315 ± 25	42 ± 2.9	117 ± 13
Stress IMI R	4	1726 ± 86	317 ± 14	37 ± 6.2	119 ± 7
Stress IMI NR	4	2068 ± 53^a^	399 ± 23^a^	53 ± 4.9	184 ± 23^a^
(b) DA and metabolites levels in striatum					
Striatum—7 weeks of stress and 5 weeks of imipramine treatment					
Treatment	*N*	[DA]	[DOPAC]	[3-MT]	[HVA]
Control	4	11,778 ± 467	1343 ± 64	482 ± 26	726 ± 84
Stress reactive	4	12,973 ± 909	1338 ± 82	508 ± 28	807 ± 84
Control IMI	4	11,594 ± 528	1420 ± 97	535 ± 43	678 ± 25
Stress IMI R	4	12,605 ± 222	1331 ± 96	344 ± 13^b^	595 ± 43
Stress IMI NR	4	13,522 ± 974	1530 ± 73	425 ± 33	837 ± 87^a^
(c) DA and metabolites levels in hypothalamus					
Hypothalamus—7 weeks of stress and 5 weeks of imipramine treatment					
Treatment	*N*	[DA]	[DOPAC]	[3-MT]	[HVA]
Control	4	349 ± 28	36 ± 4.0	10 ± 2.9	26 ± 4.1
Stress reactive	4	384 ± 30	52 ± 4.1^b^	13 ± 0.6	27 ± 4.9
Control IMI	4	358 ± 26	47 ± 1.4	21 ± 2.9	29 ± 5.3
Stress IMI R	4	373 ± 25	47 ± 5.5	13 ± 4.5	22 ± 2.2
Stress IMI NR	4	425 ± 46	58 ± 7.6	15 ± 2.6	33 ± 4.9^a^

Data represent mean ± S.E.M

^a^*p* < 0.05 indicates the statistical significant between stress IMI NR vs stress IMI R groups

^b^*p* < 0.05 indicates the statistical significant between stress vs control groups

## Discussion

### CMS and response to antidepressant treatment

Following the CMS procedure, a reduced sucrose intake was recorded in a selection of animals in response to stress stimulus and treatment with imipramine (IMI) returned these animals to a normal level of sucrose consumption (IMI-R). Additionally, this model allowed for the identification of the animals who did not respond behaviourally to antidepressant treatment (IMI-NR). The percentage of animals that is IMI-NR is usually approximately 30% of the tested animals (Faron-Górecka et al. 2014; Żurawek et al. 2015; Faron-Górecka et al. 2017). This result highlights an advantage of this animal model of depression (for review: Willner 2016), which is the expression of a treatment-resistant model of depression, as is frequently encountered in patients in the clinic.

### CMS model reflects the dynamic process of brain response to stress stimuli

One of the goals of the present study was to find a marker of drug resistance. The involvement of neuropeptides in mental disorders and in the mechanism of action of drugs has been postulated. In our previously published studies, we have demonstrated a significant negative correlation between basal prolactin levels (i.e., before the CMS procedure) and the behavioural response to IMI administration (Faron-Górecka et al. 2017). Recently, we also highlighted the role of sst_2_R in the stress reaction (Faron-Górecka et al. 2016; Faron-Górecka & Szafran-Pilch 2016). In the present study, we investigated the role of sst_2_R in response to IMI treatment following an extended period (7 weeks) of the CMS protocol. Following this experimental paradigm, increased [^125^I]Tyr^3^-Octreotide binding was observed in all brain regions involved in the stress reaction. These results are contrary to the effect of a shorter period of the CMS protocol (2 weeks), which resulted in a decrease in sst_2_R binding in the studied brain regions (with the exception of the MHb, where an increase in sst_2_R binding was observed, Faron-Górecka et al. 2016). However, the opposing effects of 2 and 7 weeks of the CMS protocol are not surprising. In our previously published papers, we observed dynamic changes that were dependent on the duration of stress at the levels of prolactin (Faron-Górecka et al. 2014). Additionally, the alterations in the dopamine D_2_ receptor binding were dependent on the duration of the CMS protocol (Żurawek et al. 2013). The observed fluctuations were observed not only at the level of receptors or neuropeptides but also at the microRNA (miRNA) level. Using the CMS protocol and studying the differences between stress-reactive and stress-resilient groups of animals, we observed fluctuations in miRNA 16 depending on the duration of the stress stimuli (Żurawek et al. 2016). It appears that the CMS model reflects the dynamic process of the brain response to stressful stimuli, which can further contribute to the development of depression.

### Role of medial habenula nucleus in the stress response

An interesting result in this study is the increase in the binding to sst_2_R in the MHb. The habenula is a small, evolutionarily conserved brain structure that plays a central role in aversive processing and is hypothesised to be hyperactive in depression, contributing to the generation of symptoms such as anhedonia (Lawson et al. 2017; Liu et al. 2017). Recently, it has been shown that dorsal MHb-lesioned mice exhibit shorter immobility time in the tail suspension test, another model of depression. Dorsal MHb-lesioned mice also display increased vulnerability to the induction of learned helplessness (Hsu et al. 2016). Statistically significant changes in the sst_2_R binding in this structure are the result of stress, whereas IMI, irrespective of the response to the treatment, produced a normalisation of this effect. Thus, it appears that the MHb is a stress-sensitive region that may be an interesting site for the study of stress resilience rather than treatment resistance (due to the lack of differences between the IMI-R and IMI-NR groups). Analogous changes in sst_2_R binding in the stress-reactive group were observed in the BLA and MeAD. Increased sst_2_R binding can be correlated with data observed by Nanda et al. (2008), who reported that rats respond to acute ferret exposure with a significant increase in fearful and anxious behaviours that are accompanied by robust amygdala activation and an increase in the expression of mRNA encoding sst_2_R within the amygdala and anterior cingulate cortex. Authors concluded that this data may represent one mechanism by which psychological stress is associated with adaptive and maladaptive behavioural responses (Nanda et al. 2008). In our studies, we also observed an increase in sst_2_R binding in the cingulate and primary cortex, which may also be relevant to the data described above. Interestingly, within this structure, we observed a statistically significant decrease in sst_2_R binding in the group of animals that were non-responsive to IMI treatment compared to the sst_2_R binding in the rats that behaviourally responded to the drug. Because it has been shown that antidepressants increase the level of SST in the PFc (Pallis et al. 2009), the reduction of SST receptors in the rats that were subjected to CMS and did not respond to IMI may indicate the involvement of SST receptors in the mechanisms of drug resistance in these animals. Moreover, it appears that the brains of animals that did not react behaviourally to IMI treatment are more sensitive to the drug treatment. After long-term CMS, increased sst_2_R binding was observed, while IMI treatment reversed this effect to the level of control in the IMI-R group. In drug-resistant animals, the reaction to IMI treatment is somehow excessive, and a statistically significant decrease in sst_2_R binding was observed. A similar effect was observed in structures in the basal ganglia. This effect is relevant to the observation that refractory depression is associated with disrupted functional connectivity mainly in thalamo-cortical circuits (Lui et al. 2011).

### Potential interaction of two receptors: sst_2_R and D_2_R

Recently, an association between thalamic hyperactivity with treatment-resistant depression and a poor response in early treatment for major depression has been shown (Yamamura et al. 2016). Long-term CMS (7 weeks) as well administration of IMI (5 weeks) caused an increase in the binding of [^125^I]Tyr^3^-Octreotide in the striatum, especially in the medial part, as well as in the NAcc. SST is synthesised in the nuclei of the basal ganglia (e.g., the striatum and NAcc), and the dysregulation of SST is implicated in motor and affective disorders (Brownstein et al. 1975; Vincent and Johansson 1983). However, cortical and subcortical SST has been implicated in the pathophysiology of psychiatric disorders (Rubinow 1986). Because the striatum and NAcc are reported to contain both SST and its receptors, it is possible that SST regulates dopaminergic function in these brain areas (Ikeda et al. 2012). It has been shown that SST infusion in the striatum leads to increased DA levels, however without changes in the DA metabolites: HVA and DOPAC (Thermos et al. 1996; Hathway et al. 1998; Marazioti et al. 2008). The sst_2_R appears to be responsible for the actions of SST on DA release and dopamine-mediated behaviours (Raynor et al. 1993; Thermos et al. 1996; Hathway et al. 1999). Thus, the observation of increased sst_2_R binding after long-lasting stress in the striatum or NAcc can result in the regulation of the dopamine D_2_R. Using [^3^H]Domperidone binding analysis, we observed a significant decrease in the expression of D_2_R in the tested brain regions and this effect was only significant in the group of animals that behaviourally reacted to stress by reducing their sucrose intake. This finding remains in agreement with our previous work, which demonstrated a regulation of D_2_R expression in response to stress: CMS decreased dopamine D_2_R mRNA expression and receptor density in the mesoaccumbens circuit in stress-reactive animals (Puglisi-Allegra et al. 1991; Papp et al. 1994; Dziedzicka-Wasylewska et al. 1997; Cabib et al. 1998; Zhu et al. 2010; Żurawek et al. 2013). Conversely, D_2_R density increased after chronic antidepressant treatment, which supports the potential involvement of D_2_R in antidepressant efficacy (Gershon et al. 2007; Dunlop and Nemeroff, 2007). In the present study, using [^3^H]Domperidone, we observed an increase in D_2_R binding upon chronic IMI treatment but this effect did not reach statistical significance. It has been shown that chronic desipramine (DMI) treatment results in an exaggerated somatostatin-induced increase in dopamine levels, specifically in the NAcc. Whereas, acute DMI treatment had no effect compared with saline-treated rats. Basal concentrations of DA and its metabolites were not shown to be influenced by either chronic or acute treatment of DMI in either brain area. These results demonstrate that SST can regulate DA release in the NAcc and striatum (Pallis et al. 2006). Likewise, it has been reported that DA administration regulates SST release (Rodriguez-Sanchez et al. 1997) and that selective DA receptor agonists increase SST receptor density in the striatum (Izquierdo-Claros et al. 1997). In our study, we measured the levels of DA and its metabolites in the PFc, striatum, and hypothalamus of rats that had been subjected to CMS. Mesolimbic and mesocortical DA is thought to play a role in the processing of rewards; however, other studies also demonstrate that DA release occurs in response to aversive stressful stimuli (Pruessner et al. 2004). The observed decrease in D_2_R binding in the stress-reactive group had no direct impact on the DA level in the striatum. Notably, in this group of animals, we observed increased sst_2_R binding. This may indicate that the D_2_R does not undergo conventional internalisation, but rather indicates the potential interaction of these two receptors, the sst_2_R and the D_2_R. The interaction between the dopaminergic and somatostatinergic systems is considered to play a potential role in mood regulation. Our previous studies have shown that D_2_R and sst_5_R heterodimers can be considered as potential mediators of the effect of antidepressants, as the heterodimerization of these receptors occurs in native brain tissue as well as in primary striatal neuronal cultures where receptors are expressed at physiological levels. Moreover, antidepressant drugs promote the formation of these heterocomplexes in the mouse striatum (Szafran-Pilch et al. 2017). Chronic administration of antidepressants influences the release of both these neurotransmitters (Pallis et al. 2009; Pallis et al. 2006; Pallis et al. 2001). The changes observed in the binding of sst_2_R and the level of DA in the groups of animals that were subjected to long-term CMS indicate that the stress context itself changes the effect of the antidepressant drug. Our results indicate a significant role of stress in the level of DA in the PFc and the striatum, with a statistically significant effect of stress on 3-MT release in the striatum and an interaction of stress and IMI treatment. However, these results depend of the behavioural reaction to IMI treatment. Exposure to a single stress stimulus, such as a restrain session, promotes an increase in the expression of DA in the PFc (Abercrombie et al. 1989). Using an alternative stress protocol, it has been demonstrated that animals exposed to CVS (chronic variable stress) exhibit a larger increase in cortical DA release in response to the restrain events than rats that were not exposed to a prior CVS regime (Cuadra et al. 1999). Similarly, in the striatum, it has been demonstrated that reduced reactivity toward noxious stimuli in animals following chronic stress corresponds with a reduced level of extracellular DA in the NAcc (Gambarana et al. 1999). Moreover, it has been demonstrated that, in some mice strains, exposure to an acute stressor induces an increase in DOPAC accumulation as well as a pronounced reduction of DA in the NAcc, while in other mice strains, these variations are less pronounced or entirely absent (Shanks et al. 1991) suggesting a close relationship between genetic or epigenetic variations and the sensitivity of the mesolimbic system and behavioural alterations which are produced by acute or chronic stress (Ventura et al. 2001; Pani et al. 2000). However, it has been demonstrated that sst_2_R functionally influence the physiology of the globus pallidus (GP) and modulate the locomotor activity of the rat. Activation of this receptor modulates the GP-striatum circuity and increases DA levels in the striatum (Marazioti et al. 2008). This data can be related to our data, suggesting an association between DA levels and sst_2_R binding. Our data revealed that IMI treatment influenced the level of DA and its metabolites (3-MT and HVA) in the PFc. However, Cuadra et al. (2001) did not show changes in DA in the frontal cortex after antidepressant drugs treatment. These authors demonstrated that repeated administration of antidepressants blocked the sensitised DA output in response to restrain following CSV exposure (Cuadra et al. 2001). An interesting finding of our study is the decreased level of DA in the PFc that was observed in the IMI-R group of animals, while the IMI-NR group showed DA levels comparable to those of the stressed group (Table 2). This suggests that animals that do not respond to IMI have impaired regulation of DA biosynthesis, providing further evidence that this group is overactive.

### Sst_2_R level is regulated by CMS in PVP

The increased level of sst_2_R binding observed in the PVP following stress or IMI treatment is another interesting finding of this study. It has been shown that the PVP plays a role in the regulation of stress and negative emotional behaviour (Hsu et al. 2014). Located in the dorsal midline thalamus, the PVP is heavily innervated by neurotransmitters and is the only thalamic nucleus that is connected to the group of structures comprising the amygdala, NAcc, and infralimbic/subgenual anterior cingulate cortex (sgACC). These neurotransmitter systems and structures are involved in regulating motivation and mood and display abnormal functioning in several psychiatric disorders including anxiety, substance use, and major depressive disorders. Furthermore, rodent studies show that the PVP is consistently and potently activated following a variety of stressors and has a unique role in regulating responses to chronic stressors. These findings provide compelling reasons to study the PVP in relation to stress and negative emotional behaviour and for including the PVP in the neural pathways involved in stress-related psychiatric disorders (Hsu et al. 2014).

## Lack of involvement of hippocampal sst_2_R in treatment resistance depression

In all of the parts of hippocampus that were studied, we observed the significant impact of treatment and stress on sst_2_R binding. It has been demonstrated that both hippocampal sst_2_ and sst_4_ receptors selectively inhibit stress-induced HPA axis activation but mediate anxiolytic and antidepressive effects through distinct mechanisms (Prévôt et al. 2017). In our study, we observed a decrease in the binding of sst_2_R in all groups, regardless of the behavioural effect following IMI treatment. This may indicate a lack of involvement of sst_2_R in hippocampal structures in the treatment resistance depression that was induced by using CMS.

## Conclusion

The obtained results indicate an involvement of sst_2_R in CMS, which is a widely used animal model of depression. In addition, we demonstrate a role of this receptor in response to treatment with IMI; the group of animals that is not responsive to IMI is more sensitive in this context, which is manifested by excessive regulation of the expression of the sst_2_R receptor and level of DA. The results obtained in sst_2_R and D_2_R binding assays, together with the measurement of the levels of DA and its metabolites in the brain, indicate an involvement of these two receptors in key brain structures involved in the response to chronic stress and antidepressant therapy sensitivity. In addition, we demonstrate that the primary cortex, cingulate cortex, striatum, and NAc are brain regions that are significant in this context.
